# Combined azelastine fluticasone nasal spray versus fluticasone propionate nasal spray in postoperative management of central compartment atopic disease (CCAD)

**DOI:** 10.3389/fphar.2026.1732886

**Published:** 2026-02-05

**Authors:** Samy Elwany, Ahmed Aly Ibrahim, Mona Elwany, Nihad Tousson-Abouelazm, Amr Kholief

**Affiliations:** 1 Department of Otolaryngology, Alexandria Faculty of Medicine, Alexandria, Egypt; 2 Department of Pathology, Medical Research Institute, Alexandria, Egypt; 3 Department of Clinical Pharmacology, Faculty of Medicine, Alexandria University, Alexandria, Egypt

**Keywords:** azelastine, CCAD, fluticasone, nasal antihistamines, nasal polyps, nasal steroids

## Abstract

**Background:**

Central compartment atopic disease (CCAD) is a subtype of chronic rhinosinusitis with nasal polyps (CRSwNP), distinguished by polypoid changes in the middle turbinates, superior nasal septum, and/or superior turbinates.

**Objective:**

The aim of this study was to compare the efficacy of combined azelastine/ fluticasone propionate spray (Azeflu) with fluticasone propionate nasal spray (FP) for management of CCAD after endoscopic sinus surgery.

**Methods:**

The present work is a prospective, randomized, double-blinded study that included 48 patients, who underwent endoscopic surgery for refractory CCAD. The patients were randomly enrolled into two groups using simple randomization. Group A patients received FP (50 μg per spray) twice daily, while group B patients received Azeflu spray (137/50 μg, Aze/FP per spray) twice daily. Outcome measures included clinical assessment (SNOT-22), Lund Mackay radiologic score (LM), total serum IgE, nasal mucosa eosinophil count, and polyps score, and need for revision surgery. The results were collected before surgery as well as 6 and 12 months after surgery.

**Results:**

SNOT-22 scores, total serum IgE, and nasal mucosa eosinophil count decreased significantly in both groups at the end of follow-up period. The decrease was significantly greater in group B patients who received the combined spray. On the other hand, there was a modest decrease in LM scores at 6 and 12 months after surgery in both groups. The frequency of polyps recurrence was significantly less in group B than in group A.

**Conclusion:**

The present research shows that nasal sprays may effectively reach the target areas in the central compartment after surgery, potentially benefiting from the widened post-surgical nasal corridor. Combined steroid/H1 blocker sprays provided better postoperative control of CCAD than steroid sprays. The synergistic action of H1 blockers in the combined sprays confirms the association between CCAD and nasal allergy and encourages us to consider it as a first line treatment for the disease.

## Introduction

Central Compartment atopic disease (CCAD) is a distinct type of chronic rhinosinusitis (CRS) representing allergy process affecting the central nasal compartment and resulting in polypoid changes involving middle turbinates and adjacent nasal mucosa (DelGaudio et al., 2017). The polypoid changes may then progress from medial to lateral to the sinuses as a simple obstructive phenomenon. This pattern of CRS is distinct from the more diffuse chronic sinusitis with nasal polyps (CRSwNP) and requires allergy management as a core component of the treatment protocol (Wilson et al., 2014).

The association between CRSwNP and allergy has been comprehensively studied but is still controversial as there is a modest level of evidence that supports a link between the two entities (Wilson et al., 2014). On the other hand, CCAD is more evidently associated with atopic diseases (White et al., 2014; Hamizan et al., 2018). In 2017, DelGaudio et al. defined CCAD as a variant of chronic rhinosinusitis strongly associated with allergy and demonstrating edema and polypoid changes of the central sinonasal compartment (DelGaudio et al., 2017).

Most patients with CRSwNP have Type 2 (Th2) pattern of inflammation characterized by high levels of eosinophils, mast cells, IgE, basophils and T-helper (Th2) cells. In addition, multiple cytokines; interleukin-4, interleukin-5 and interleukin-13 have been found to drive the immunological pathways in CRSwNP (Dennis et al., 2016). CCAD, on the other hand, represent a local immune response related to antigen contact in the central nasal areas that are normally most exposed to inhaled air (Kong et al., 2022). In the study by White et al., all of 16 patients with isolated polypoid middle turbinate tested positive for both seasonal and perennial allergens (White et al., 2014). The authors also pointed to the likely role of the middle turbinate as a protective structure that prevents inhaled particles from entering the middle and superior meatus. Hamizan et al., reported that polypoid middle turbinate had an excellent positive predictive value for the presence of inhalant allergy (Hamizan et al., 2018).

In accordance with international recommendations for CRSwNP, all patients initially received a trial of symptomatic medical therapy consisting of daily intranasal corticosteroid sprays (INCS) combined with regular saline nasal irrigations for a minimum of 8–12 weeks, consistent with guideline-recommended first-line therapy. Patients whose symptoms and polyp burden persisted after this period were classified as “refractory to medical management” and subsequently referred for endoscopic sinus surgery (ESS). However, ESS alone is not curative and recurrence of the polyps after surgery is a well-known stubborn problem.

Topical nasal sprays hardly reach the central nasal compartment in the presence of polyps before surgery. This situation markedly changes after surgery when the corridors to the central nasal area and middle turbinates are widely open. This encouraged the use of intranasal steroid sprays and irrigations after endoscopic sinus surgery to reduce sinonasal mucosal inflammation and slow down recurrences of the polyps (Rank et al., 2023a; Rank et al., 2023b). On the other hand, topical antihistamines were not routinely used in the postoperative management of CRSwNP because of their limited efficacy apparently because of the weak controversial link between it and nasal allergy (Marcus et al., 2019) (Lilly et al., 2024).

CCAD, unlike CRSwNP, is closely linked with atopy, and antihistamines may help in managing the disease and reducing polyp recurrence. The intranasal formulation of azelastine HCl (AZE, an antihistamine) and fluticasone propionate (FP, a corticosteroid) in a single spray was originally introduced in the United States in 2016 (Prenner, 2016). Azelastine as H1 receptor antagonist reduces eosinophil survival and inhibits the release of pro-inflammatory mediators and cytokines by nasal epithelial cells (Mullol et al., 2011). When combined with fluticasone propionate, azelastine showed a synergistic anti-inflammatory effect leading to improvement of symptoms of nasal allergy and has been recommended for moderate and severe allergic rhinitis (Luo et al., 2015) (Roca-Ferrer et al., 2018; Klimek et al., 2021). However, to our knowledge postoperative use of topical antihistamine/steroid combinations like azelastine/fluticasone for CCAD has not been previously investigated.

Herein, the present prospective randomized study was designed to compare the effectiveness of fluticasone nasal spray versus the combined azelastine/fluticasone spray (Azeflu) in improving the patients’ symptoms and preventing recurrence of the polyps following ESS for CCAD.

## Patients and methods

**FIGURE 1:**
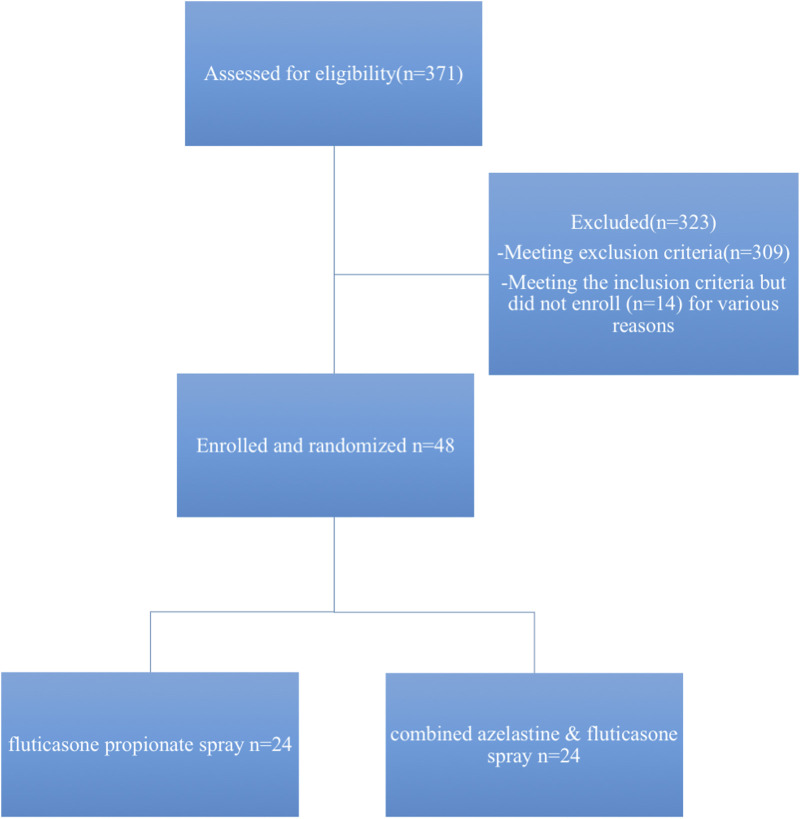
Patient flow.

Patient cohort: A total of 48 patients with CCAD were enrolled in this study. Enrolment of patients began in March 2018 and was completed in February 2025. A total of 62 patients fulfilled the inclusion criteria of whom 48 patients completed the treatment protocol (Figure 1).

Inclusion criteria included: the presence of endoscopic and radiological evidence of CCAD with nasal polyps that persisted despite standard medical management. Refractoriness was determined based on the patient’s clinical history, physical examination, and radiographic findings after completing an adequate trial of guideline-recommended therapy. Patients who demonstrated this persistent disease despite appropriate medical treatment were considered suitable candidates for endoscopic sinus surgery and were therefore eligible for enrollment. All patients had positive allergy testing.

Exclusion criteria included: other subtypes of chronic rhinosinusitis (CRS), fungal rhinosinusitis, cystic fibrosis, mucociliary disorder, aspirin-exacerbated respiratory disease. (AERD), systemic steroid dependence for any medical condition, patients with uncontrolled allergy requiring oral steroids or immunotherapy, and previous surgery. Patients who discontinued the follow-up or could not complete the trial were also excluded from the study.

Study design: This randomized, double-blinded, prospective study was conducted at a tertiary referral hospital. A diagnosis of CRSwNP was made based on criteria established by the International Consensus Statement in Allergy and Rhinology: Rhinosinusitis and the European Position Paper on Rhinosinusitis and Nasal Polyps (Fokkens et al., 2020; Orlandi et al., 2016).

The study was approved by the university ethics committee IRB NO:00012098, and all procedures performed were in accordance with the ethical standards of the institutional research committee. Written informed consent was obtained from all participants included in the study prior recruitment.

Following endoscopic sinus surgery (ESS) the patients were randomized into 2 groups. A simple randomization method was used: Group A (24 patients) used fluticasone propionate spray (Flixonase (GSK), 50 μg FP per spray), Group B (24 patients) used combined azelastine and fluticasone spray (Azelast Plus (Hikma), 137/50 μg Aze/FP per spray).

Throughout the study, all patients within each group received the same formulation for the assigned medication. The patients were instructed to spray 2 puffs in each nostril twice per day 10 min after cleaning the nose with saline washes. The compositions of the used sprays were hidden to the investigators and patients as well.

None of the patients received oral steroids or oral antihistamines. Patients who needed any of these medicines or did not complete the follow-up were excluded from the study (Janežič et al., 2017).

### Study workup

Pre-operatively, patients underwent clinical, radiological, endoscopic, and laboratory assessments. Clinical (subjective) assessment of patients was done using the Sino-Nasal Outcome Test-22 for quality of life of the patient (SNOT-22) questionnaire, which includes evaluation of olfactory function as one of its symptom items (Elwany et al., 2017), radiological assessment was done by non-contrast, bone and soft tissue window Computed Tomography (CT) examination and disease severity was graded according to Lund-Mackay radiologic grading system (LM) (Hopkins et al., 2007). Endoscopic assessment was quantified using the polyps score which is a clinical endoscopic score to define the extent of the polyps (0 = none, 1 = confined to central nasal compartment, 2 = extending beyond central nasal compartment). The Modified Lund-Kennedy endoscopic score could not be used since it included many non-applicable items (Psaltis et al., 2014).

Laboratory assessment included allergy testing, measurement of total serum IgE levels (IU/ml), and tissue eosinophil count. Epicutaneous allergy testing was performed using skin prick testing. Evaluation was done using a panel of ALK standard extracts of pollens, pollen mixes, epidermal extracts and mites. In brief, the test was performed on the volar forearm, the panel of allergens were applied in a 50% glycerin solution along with a negative (glycerin) control and a positive (histamine) control. Wheal responses were measured after 15 min and a reaction >3 mm greater than the negative control was considered positive.

Serum-specific IgE antibodies for the four aeroallergen mixes (pollens, pollen mixes, epidermal extracts and mites) were assessed via automated immunoassay. A serum specific IgE level of greater than 0.35 KU/L for any of these allergens was considered a positive result and the patient was considered a topic.

Polyp tissues were obtained during endoscopic surgery and processed using standardized procedures. Paraffin-embedded samples were sectioned at 4/µm thickness and were stained with hematoxylin and eosin stain (H&E) and examined with light microscopy. Tissue eosinophil counts were calculated by 2 independent pathologists blinded to the patients’ group. The eosinophil count (n/HPF) was recorded as the mean of the counts for 10 nonoverlapping high-power fields in the lamina propria at ×400 magnification using a bright-field light microscope (Lou et al., 2015; Yu et al., 2021).

### Operative technique

All patients had undergone endoscopic sinus surgery under general anesthesia. The extent of surgery was tailored according to the extent and severity of the disease. All polyps were resected with a shaver and the sinuses were conservatively opened as needed. The polypoid parts of the middle turbinates were conservatively trimmed. Small Merocel packs were placed at the endow surgery.

### Postoperative workup

Patients were placed on postoperative antibiotics for 10 days. Regular weekly postoperative follow up was arranged to provide debridement, suction of crusts and cleaning of sinonasal cavity for 2 months. The patients were then followed up monthly for the rest of the 12-month follow up period.

The postoperative workup was done at 6 months and was repeated at 12 months. Counting of nasal eosinophils was repeated only at the end of follow up period. Tiny biopsies from mucosa in the central compartment or polyps in case of recurrences under topical anesthesia and processed as mentioned before.

Patients in the two groups received the designated nasal spray according to the treatment protocol. Compliance of patients to medication intake was assessed by the Medication Adherence Questionnaire; Morisky Medication Adherence Scale-8 (MMAS) (Janežič et al., 2017).

Postoperative evaluation included subjective assessment and objective assessment. Subjective assessment was performed using the SNOT-22. Objective assessment included radiological evaluation using the Lund-Mackay scoring system (LM),measurement of total serum IgE levels, tissue eosinophil count and endoscopic evaluation using the polyp score: score 0 = no polyps, score 1 = polyps confined to the central compartment of the nose, score 2 = polyps extending beyond the central compartment.

The primary outcome measures were the improvement of patients’ symptoms (SNOT-22) and the frequency of polyps recurrence. The secondary outcome measure was the need for revision surgery.

### Statistical analysis

The sample size was estimated according to the standard parameters (Bhalerao and Kadam, 2010). Data were fed to the computer and analyzed using IBM SPSS software package version 20.0. (Armonk, NY: IBM Corp) Qualitative data were described using number and percent. Normality of continuous variables was evaluated using the Shapiro–Wilk test, supplemented by visual inspection of histogram plots. Variables demonstrating a normal distribution were described using range (minimum and maximum), mean, standard deviation. The Chi-square test, Student’s t-test, and Wilcoxon signed ranks test were used. The tests were performed with 95% threshold of significance and a Type I error rate (or alpha) of 5%.

## Results

A total of 48 patients with CCAD were enrolled in this study. Enrolment of patients began in March 2018 and was completed in February 2025. A total of 62 patients fulfilled the inclusion criteria of whom 48 patients completed the study protocol. The patients were randomized into 2 groups according to the type of nasal spray used after surgery. Group A included 24 patients who received fluticasone propionate spray, and Group B included 24 patients who received azelastine hydrochloride/fluticasone propionate spray. All patients used saline washes before spraying the nasal cavities. The flow chart of participants was shown in Figure 1.

**FIGURE 2:**
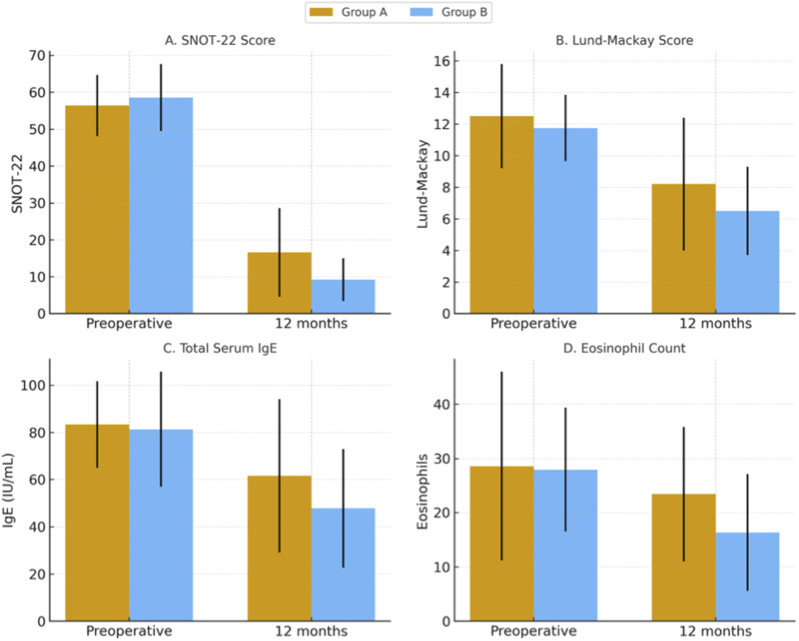
Comparison of preoperative and 12-month postoperative between Group A (Fluticasonepropionate) and Group B (Azelastine/Fluticasone). Bars represent mean values with error bars indicating 95% confidence intervals (calculated as 1.96 × standard error).

Basic patients’ demographic data are shown in Table 1. There was no significant difference between the 2 groups as regard their demographic characteristics: age, sex, smoking, associated asthma, or any other confounding variables. Similarly, both groups had no significant difference as regards their preoperative data: SNOT-22, Lund-Mackay score, total serum IgE, tissue eosinophil count, and polyp grading (Tables 2–6). Figure 2 summarizes the results.

**TABLE 1:** Demographic data and patient characteristics.

​	A (n = 24) (Fluticasone propionate)	B (n = 24) (Azelastine/Fluticasone)	P
	Number (%)	Number (%)	
Sex			
Male Female	17 (71.8) 7 (29.2)	19 (79.2) 5 (20.8)	P = 0.5409
Age (years)			
Age (years) Min - max Mean (SD)	18.0–64.0 39.44 ± 18.28	20.0–59.0 40.28 ± 15.92	P = 0.6470
Smoking			
Yes No	6 18	4 20	P = 0.4771
Asthma			
Yes No	3 21	5 19	P = 0.4385

**TABLE 2:** Comparison between the two groups according to SNOT-22 score.

​	A (n = 24) (Fluticasone propionate)	B (n = 24) (Azelastine/Fluticasone)	​
Preoperative			
Min-Max Mean ± SD	29–62 56.4 ± 8.3	32–61 58.542 ± 9.1	P_0_ = 0.463
6 months postoperative			
Min-Max Mean ± SD	6–32 14.6 ± 8.3	5–29 8.1 ± 7.2	P_1a_ = 0.043^a^ P_1b_ = 0.031^a^
P_1_ = 0.023^a^			​
12 months postoperative			
Min-Max Mean ± SD	8–35 16.6 +/12.00	6–31 9.2 ± 5.8	​
P_2_ = 0.014^a^			​

P_0:_ p value for preoperative comparison of the two groups.

P_1:_ p value for 6-month postoperative comparison of the two groups.

P_1a:_ p value for preoperative and 6-month postoperative comparison of group A.

P_1b:_ p value for preoperative and 6-month postoperative comparison of group B.

P_2:_ p value for 12-month postoperative comparison of the two groups.

^a^ Statistically significant at p ≤ 0.05.

**TABLE 3:** Comparison between the two studied groups according to Lund-Mackay score.

​	A (n = 24) (Fluticasone propionate)	B (n = 24) (Azelastine/Fluticasone)	​
Preoperative			
Min-Max Mean ± SD	10–14 12.50 ± 3.3	11–14 11.75 ± 2.1	P_0_ = 0.236
6 months postoperative			
Min-Max Mean ± SD	6–13 8.6 ± 4.3	5–13 7.1 ± 3.6	P_1_a = 0.192 P_1b_ = 0.117
P_1_ = 0.898			​
12 months postoperative			
Min-Max Mean ± S	6–12 8.2 ± 4.2	5–10 6.5 ± 2.8	​
P2 = 0.744			​

P_0:_ p value for preoperative comparison of the two groups.

P_1:_ p value for 6-month postoperative comparison of the two groups.

P_1a:_ p value for preoperative and 6-month postoperative comparison of group A.

P_1b:_ p value for preoperative and 6-month postoperative comparison of group B.

P_2:_ p value for 12-month postoperative comparison of the two groups.

**TABLE 4:** Comparison between the two studied groups according to total serum IgE (IU/mL).

​	A (n = 24) (Fluticasone propionate)	B (n = 24) (Azelastine/Fluticasone)	​
Preoperative			
Min-Max Mean ± SD	14–198 83.35 ± 18.3	12–210 81.3 ± 24.4	P_0_ = 0.717
6 months postoperative			
Min-Max Mean ± SD	12–156 62.5 ± 13.9	10–127 49.8 ± 22.3	P_1_ = 0.035^a^
12 months postoperative			
Min. – Max Mean ± SD.	11.0–159.0 61.60 ± 32.53	10.0–123.0 47.82 ± 25.12	P_2_ = 0.032^a^
​	p_3_ = 0.022^a^	p_3_ = 0.015^a^	​

P_0:_ p value for preoperative comparison of the two groups.

P_1:_ p value for 6-month postoperative comparison of the two groups.

P_2:_ p value for 12-month postoperative comparison of the two groups.

P_3_: p value for comparing between preoperative and 12 months postoperative in each group.

^a^ Statistically significant at p ≤ 0.05.

**TABLE 5:** Comparison between the two groups according to eosinophil count.

​	A (n = 24) (Fluticasone propionate)	B (n = 24) (Azelastine/Fluticasone)	p
Preoperative			
Min. – Max Mean ± SD.	58.0–76.0 68.00 ± 17.38	59.0–85.0 70.50 ± 11.44	P_0_ = 0.467
12 months postoperative			
Min. – Max Mean ± SD.	53.0–61.0 56.5 ± 12.39	49.0–56.0 52.0 ± 10.77	P_1_ = 0.022^a^
​	P_2_ = 0.045^a^	p_3_ = 0.013^a^	​

P_0_: p value for preoperative comparison of the 2 groups.

P_1_: p value for comparing the 2 groups at the end of follow up period (12 months).

p_2_: p value for comparing preoperative and 12 months postoperative results for group A.

p_3_: p value for comparing preoperative and 12 months postoperative results for group B.

^a^ Statistically significant at p ≤ 0.05.

**TABLE 6:** Comparison between the two groups according to polyp grading.

​	A (n = 24) (Fluticasone propionate)		B (n = 24) (Azelastine/Fluticasone)		​	​
	No	%	No	%		
Preoperative						
No polyps Polyps confined to the central compartment Polyps extending beyond the central compartment	0 22 2	0 91.7 8.3	0 21 3	0 87.5 12.5	χ^2^= 0.270	P_0_ = 0.603
6 months postoperative						
No polyps Polyps confined to the central compartment Polyps extending beyond the central compartment	15 8 1	62.5 33.3 4.2	17 7 0	70.8 29.2 0	χ^2^= 1.192	P_1_ = 0.275
12 months postoperative						
No polyps Polyps confined to the central compartment Polyps extending beyond the central compartment	9 13 2	37.5 54.2 8.3	16 8 0	66.7 33.3 0	χ^2^= 5.150	P_2_ = 0.023^a^

P_0_: p value for preoperative comparison of the 2 groups.

P_1_: p value for comparing the 2 groups at 6 months postoperative.

p_2_: p value for comparing preoperative and 12 months postoperative.

^a^ Statistically significant at p ≤ 0.05.

There was a clear predilection for sensitization to perennial allergens, particularly epidermals and mites. Both groups showed significant improvement in their SNOT-22 scores at the first postoperative evaluation at 6 months after surgery (p_1a_ = 0.043, p_1b_ = 0.031). Group B showed more significant improvement than group A patients (p_1_ = 0.023). The improvement was maintained after 12 months with a greater statistically significant difference between the 2 groups (p_2_ = 0.014) (Table 2).

Although the radiologic Lund Mackay scores decreased in both groups after surgery, the decrease was not statistically significant. Also, there was no significant difference between the two groups at the end of follow-up period (Table 3).

There was no significant difference in total serum IgE levels between the two groups before surgery. The level of total serum IgE decreased from 83.35 (IU/mL) to 62.5 (IU/mL) at 6 months and to 61.6 at 12 months in group A, and from 81.3 (IU/mL) before treatment to 49.8 (IU/mL) at 6 months and 47.82 (IU/mL) at 12 months in group B, The drop was significantly greater in group B patients both at 6 months (p_1_ = 0.035) and 12 months after surgery (p_2_ = 0.032) (Table 4).

There was no significant difference between the 2 groups regarding nasal mucosa eosinophil count at the beginning of the study. The count decreased from 68 to 56.5 in group A (p_2_ = 0.045) and from 70.5 to 52.0 in group B (p_3_ = 0.013) at 12 months after surgery. The drop in the count was significantly greater in group B at the end of follow-up period (p_1_ = 0.022) (Table 5).

The polyp grading score decreased in both groups at 6 and 12 months after surgery. There was no significant difference between the two groups in the scores before surgery as well as at 6 months after surgery. However, the score was significantly less in group B at 12 months after surgery (p_2_ = 0.023) indicating significantly less polyps recurrence in group B patients (Table 6).

Three patients in group A (12.5%) and only 1 patient in group B (4.1%) needed revision surgery. The primary indication for revision surgery was polyp recurrence causing significant hyposmia or anosmia not responding to oral steroids.

## Discussion

Central compartment atopic disease is a distinct inhalant allergy-related variety of CRS that is clinically different from the more common CRSwNP (DelGaudio et al., 2017). A major pathologic difference between the 2 entities is their association with nasal allergy. While the link of CRSwNP with nasal allergy is still controversial and unclear the association between CCAD and allergy is more evident (Marcus et al., 2018) (Kariyawasam and James, 2020). The localized allergic process gives rise to polypoid degeneration of the nasal mucosa of the superior nasal septum, middle turbinates, and superior turbinates (Marcus et al., 2019).

The CCAD phenotype was first identified by White et al., in 2014, revealing an association between isolated middle turbinate polypoid edema/polyps and inhalant allergies (White et al., 2014). The report was further validated by Hamizan et al. (2018) and Brunner et al. (2017) who reported increased allergen sensitivities and a strong connection to allergic rhinitis in patients with isolated middle turbinate polyps. Later, Edwards et al. compared systemic allergen sensitivity and local allergen sensitivity in the sinonasal tissue of patients with CCAD and found that of the 15 participants, 14 were sensitive to at least 1 allergen locally in the central compartment and systemically on skin or serum testing (Edwards et al., 2022). Marcus et al. found that the prevalence of asthma is low in patients with CCAD in spite of the high prevalence of allergy in the same patients (Marcus et al., 2020).

Delivery of nasal sprays for patients with nasal polyps before surgery is frequently inefficient since little of the spray is delivered to the inflamed sinonasal mucosa in the presence of polyps. This situation changes after surgery because of the wide postoperative corridor which allows better delivery of the spray to the mucosa.

Topical fluticasone propionate has potent anti-inflammatory action on the sinonasal mucosa by reducing airway infiltration by eosinophils, mast cells, and T-lymphocytes, and by suppressing the release of adhesion molecules and pro-inflammatory genes and mediators. However, the combined use of intranasal AZE and FP in a single intranasal formulation has been reported as more effective alternative than monotherapy (Roca-Ferrer et al., 2018) and as a first-line option for moderate-to-severe allergic rhinitis (Bousquet et al., 2016).

Several clinical trials have shown superior efficacy of Azeflu in patients with allergic rhinitis with the advantages of rapid onset of action and faster relief of symptoms (Bousquet et al., 2020). *In vitro* studies comparing the pharmacokinetics of single FP formulation and Azeflu formulation have demonstrated that FP tissue penetration is significantly greater with the Azeflu formulation than with the FP formulation (Berger et al., 2020). Further research has explored the effects of Azeflu on inflammatory markers, such as cytokines and eosinophil survival, using an *in vitro* model. Azeflu demonstrated a significantly greater inhibition of IL-6 secretion compared to either FP or AZE alone, at the same drug dilution. Additionally, Azeflu showed a markedly superior, time-dependent inhibition of eosinophil survival, compared to FP or AZE, at days 3 and 4, again at the same drug dilution. These findings suggest a potential synergistic effect of the Azeflu combination (Roca-Ferrer et al., 2018).

Considering the clear association between allergy and CCAD, the combined Azeflu spray is expected to be more efficient than FP alone in controlling the allergic reaction and reducing the incidence of polyp’s recurrence after surgery. To our knowledge, this is the first randomized prospective cohort study to investigate the efficacy of combining H1-receptor antagonist (azelastine) with intranasal corticosteroid (fluticasone propionate) in postoperative management of patients with CCAD. The study aimed to assess the efficacy of the combination in reducing polyp recurrence, alleviating nasal symptoms and enhancing the quality of life of patients compared to the use of intranasal fluticasone propionate spray alone.

Both groups showed significant decrease in their SNOT-22 scores at the first postoperative evaluation at 6 months after surgery. Group B patients had lower scores than group A patients. The improvement was maintained after 12 months with a greater statistically significant improvement in group B patients.

Although the radiologic Lund Mackay scores decreased in both groups after surgery, the decrease was not statistically significant. Also, there was no significant difference between the two groups at the end of follow-up period. The modest decrease in LM scores was apparently due to minimal involvement of the sinuses in CCAD.

Total serum IgE decreased significantly at 6 and 12 months postoperatively in both groups with greater decrease in group B patients indicating the additive effect of H1 blockers (azelastine) in patients with CCAD. This additive effect was confirmed by the greater drop in nasal mucosa eosinophil count in group B patients at the end of follow-up period.

Recurrence rate of the polyps was similar in the 2 groups at 6 months postoperatively but was significantly less in group B when the patients were followed up for 12 months. This decrease in recurrence rate is explained by the reduction of the IgE-mediated allergic process in the nasal mucosa and is a further indication of the superior effect of combined sprays in patients with CCAD. In addition to attenuating IgE-mediated mast cell activation, the AZE/FP combination may also reduce inflammation through mechanisms that are not directly dependent on IgE-triggered degranulation. Azelastine has been shown to inhibit the release of multiple mast-cell mediators—including histamine, leukotrienes, and cytokines—through both IgE-dependent and non–IgE-dependent pathways, thereby exerting broader anti-inflammatory activity. Fluticasone propionate likewise suppresses a wide array of inflammatory processes by reducing cytokine transcription, decreasing eosinophil survival, and stabilizing multiple inflammatory cell types, including mast cells and basophils. Therefore, it is plausible that inhibition of other pro-inflammatory stimuli—such as mast cell mediators not directly linked to IgE-mediated degranulation—also contributed to the reduced inflammatory burden and subsequently lower recurrence observed in the AZE/FP group. This broader anti-inflammatory profile supports the clinical effect observed with the combination therapy.

Three patients in group A (12.5%) and only 1 patient in group B (4.1%) needed revision surgery. The primary indication for revision surgery was polyps recurrence causing hyposmia or anosmia not responding to oral steroids. CCAD is considerably associated with olfactory dysfunction and ESS can improve olfactory function by clearing the pathway to the olfactory mucosa (Huang et al., 2023). Careful removal of polyps from the olfactory cleft without damaging the olfactory neuroepithelium is recommended to optimize postoperative olfaction.

CCAD is a distinct inflammatory sub-type of CRSwNP with clear association with nasal allergy. Only a few studies have assessed surgical outcomes and are scarcely reported in the literature. Nasal sprays are less troublesome and easier to use than large volume steroid irrigations and the present research showed that they can effectively reach the target areas in CCAD taking advantage of the widened post-surgical nasal corridor.

The primary conclusion of this study is that although both of steroid sprays and combined steroid/H1 blocker sprays may be effectively used in CCAD after endoscopic surgery, the combined sprays provided more durable control of the disease, less frequency of polyp recurrence after surgery, and less need for revision surgery. The study further confirms the intricate association between CCAD and allergy.

Some limitations of this study must be considered. First, the sample size was rather small, although it was statistically calculated, since the disease is not as frequent as CRSwNP. Second, patients were monitored only during the duration of the study, and not on a long-term basis for better assessment of the rate of recurrence of the polyps. The present research is a single-institution study and may not accurately reflect regional influences. Multi-institutional studies that consider different regions and populations are needed to support the results of the present study.

## Data Availability

The original contributions presented in the study are included in the article/supplementary material, further inquiries can be directed to the corresponding author.
